# Bladder Perforation in the Elderly: Unraveling the Diagnostic Challenges With Magnetic Resonance Imaging

**DOI:** 10.7759/cureus.48934

**Published:** 2023-11-17

**Authors:** Frederico Silva, Mari Angela Lopes, Dilia Valente, Inês G Simões, Luísa Arez

**Affiliations:** 1 Internal Medicine, Centro Hospitalar Universitário do Algarve - Unidade de Portimão, Portimão, PRT

**Keywords:** timely recognition, diagnostic challenge, catheter-related complications, urinary catheter, bladder perforation

## Abstract

Bladder perforation, a significant urological emergency, presents a diagnostic challenge due to its diverse etiologies and varying clinical manifestations. This paper discusses a rare case of bladder perforation in an 87-year-old woman with a history of hypertension and previous stomach and uterine cancer. The patient was admitted with a urinary tract infection and subsequently experienced mild abdominal discomfort and reduced urinary output, prompting further investigation. Imaging studies revealed bladder wall thickening and ureterohydronephrosis, raising suspicion of a bladder tumor. Intriguingly, a catheter-related bladder perforation was identified through MRI. This case emphasizes the importance of considering bladder perforation as a potential complication, especially in elderly patients with indwelling catheters. Clinicians must maintain a high index of suspicion and employ appropriate diagnostic modalities to ensure timely recognition and suitable management of this rare condition.

## Introduction

Bladder perforation, marked by a breach in the bladder wall, is a medically significant condition with potentially severe consequences. It is a rare urological emergency primarily caused by blunt external trauma. This complication can also stem from iatrogenic injuries, urinary obstruction, or underlying pathological conditions. The challenge for clinicians lies in its diverse clinical presentations, ranging from subtle symptoms to life-threatening situations. Maintaining a high index of suspicion for bladder trauma is vital, especially when patients present with hematuria, suprapubic pain, peritoneum-related symptoms (rebound tenderness, guarding, rigidity, percussion tenderness, localized tenderness), ascites, urinary retention, or anuria following vesical catheterization [1].

Bladder perforation is also frequently linked to abdominal and pelvic surgeries, occurring in approximately 1 in 1000 cases. Catheterization, on the other hand, is an exceptionally rare cause of bladder injury, with only a few reported instances [2].

## Case presentation

An 87-year-old woman with a medical background of hypertension, dyslipidemia, and previously treated stomach and uterine cancers was admitted to an internal medicine ward due to a urinary tract infection.

While in the hospital, the patient experienced episodes of vomiting and weakness, prompting the need for a CT scan of the thoracic, abdominal, and pelvic regions. The examination revealed thickening of the bladder wall, particularly on its left lateral side, displaying enhanced intensity and unclear boundaries. This thickening extended to the area around the left ureteral orifice, resulting in ipsilateral ureterohydronephrosis. These findings, after discussion with our urology colleagues, prompted the decision to conduct an MRI to further investigate the potential presence of a bladder tumor.

While awaiting an MRI, the patient underwent urethral catheterization to monitor urine output. Following catheterization, a yellowish and cloudy liquid emerged in the collection bag, resembling urine. There were no concerns about the catheter's incorrect placement, so the liquid obtained was not subjected to testing. By the fourth day of hospitalization, she began experiencing mild abdominal discomfort. Upon symptom review, it was noted that her urinary output had decreased in the past day (150 mL in the last 24 hours). Initially, there was concern about dehydration or a potential urinary tract infection related to the catheterization the previous day. She denied any fever, external injuries, blood in urine, or difficulties in emptying her bladder. The patient reported no recent changes in bowel or bladder habits but mentioned additional urinary losses outside the catheter since the procedure. The laboratory parameters showed a benign progression. The patient displayed no signs of infection or intra-abdominal complications.

**Figure 1:**
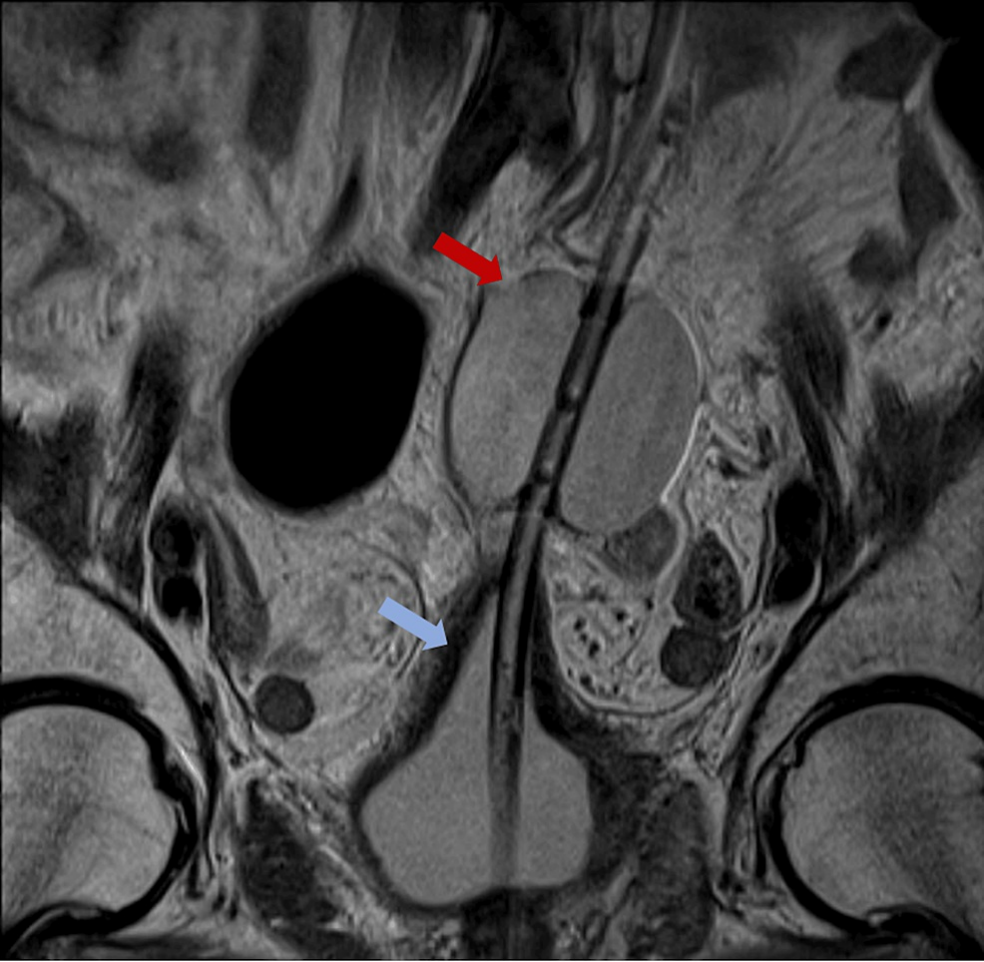
Coronal view in MRI of the bladder perforation. The red arrow points to the catheter balloon; the blue arrow points to the deformed bladder.

**Figure 2:**
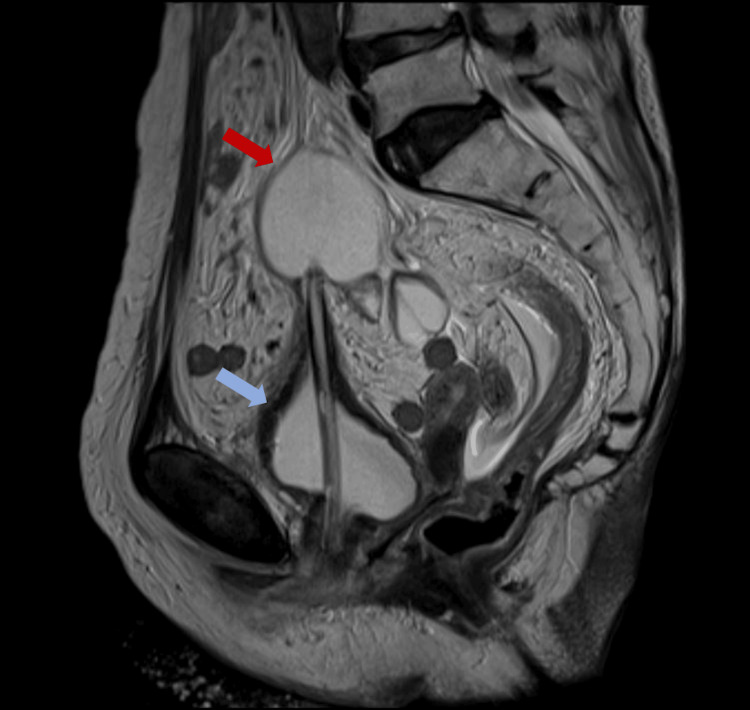
Sagittal view in MRI of the bladder perforation. The red arrow points to the catheter balloon; the blue arrow points to the deformed bladder.

Incidentally, the scheduled MRI to investigate the potential bladder cancer was conducted on the same day. The images revealed a bladder wall perforation caused by a catheter, with the catheter balloon positioned above the bladder (Figures 1-2).

The urology department was consulted; they then repositioned the catheter under ultrasound guidance with clear urine output observed. After repositioning, the catheter presented itself as an echogenic tube extending from the urethra into the bladder. The catheter's balloon could be seen as an echogenic ovoid structure within the bladder lumen. A conservative approach was chosen. Antibiotic therapy (piperacillin-tazobactam) already started for the urinary tract infection was continued. Since the patient's condition remained benign, additional treatment was deemed unnecessary. No additional CT scan was requested. Subsequent urine laboratory analyses in the following days returned normal results, and the urinary output corresponded with the stable hemodynamic condition of the patient.

No significant incidents occurred during the hospital stay. The patient stabilized and was subsequently discharged; follow-up with the urology department was scheduled on an outpatient basis.

## Discussion

Bladder injuries commonly result from high-impact trauma, but there are reported cases of bladder rupture without any apparent traumatic cause. Such ruptures have been linked to malignant diseases, urinary flow blockage, or a combination of factors. Studies have indicated bladder injuries related to catheterization, specifically with chronic intermittent catheterization or prolonged use of indwelling catheters, rather than single placements like Foley catheters [3]. In this instance, the prior history of uterine cancer radiotherapy might have played a role in weakening the bladder wall, potentially contributing to this event.

Perforation of the urinary bladder within the peritoneal cavity commonly presents as abdominal pain accompanied by guarding due to peritonitis [4]. In this particular case, following an uncomplicated catheterization (as reported by the nursing staff), there were no concerns regarding the presence of bladder perforation. It's important to note that the patient didn't suffer from intense abdominal pain. This underscores the necessity of staying vigilant for catheterization-related complications. This vigilance is crucial because usually, no further tests are performed to confirm proper positioning, leading to frequent oversight of urologic complications [5].

In this case, bladder perforation was identified through an MRI, although CT scans are typically the standard choice for assessing abdominal injuries. Yet, alternative studies propose that retrograde cystograms or ultrasounds might be more effective in diagnosing bladder injuries. Properly choosing between CT scans and cystograms is crucial for accurate diagnosis in cases of bladder injuries [6].

Typically, intraperitoneal urinary bladder perforation necessitates urgent surgery to prevent life-threatening peritonitis [4]. Nevertheless, in this particular instance, after consulting with urology, a conservative approach to management was selected. After the urinary catheter was repositioned, there was an immediate return to clear urine output, and no signs of hematuria were detected. Moreover, in the days following this incident, no issues related to gastrointestinal, cardiovascular, or infectious dysfunction were observed.

## Conclusions

In conclusion, this case highlights a rare occurrence of bladder perforation. As the use of indwelling catheters becomes more prevalent, clinicians must remain vigilant about recognizing the signs and symptoms of bladder perforation in patients with such catheters. Awareness of this potential complication is crucial for timely diagnosis and appropriate management.

Given the rarity of finding this pathology in MRI, it is considered relevant for its pedagogical value.
